# Construing Complex Referentiality in Interspecies Interaction: Embodiment and Biosemiotics

**DOI:** 10.3390/ani15233430

**Published:** 2025-11-28

**Authors:** Rea Peltola, Marine Grandgeorge

**Affiliations:** 1 CRISCO (Centre de Recherches Inter-Langues sur la Signification en Contexte)—UR4255, Unicaen, Normandie University, F-14032 Caen, France; 2 EthoS (Éthologie Animale et Humaine)—UMR 6552, Centre National de la Recherche Scientifique (CNRS), University Rennes, Normandie University, F-35000 Rennes, France; marine.grandgeorge@univ-rennes.fr

**Keywords:** interspecies interaction, child-pet play, embodiment, biosemiotics, referentiality, complex semantics

## Abstract

This paper investigates language use in interspecies interaction. The aim is to characterize the variation in children’s pet-oriented speech and to explore how semantic complexity is treated. The analysis first presents quantitative observations on properties of talk and the ongoing activities. The dataset includes 19 video recordings presenting cats, dogs, and 6–12-year-old French-speaking children in an ecological context. The analysis showed that children spoke more to dogs than to cats. We also found a strong correlation between play activity and the amount of talk. We then observed that, just like adults in the previous literature, children use short utterances when talking to pets. This may indicate syntactic simplification. The second part of the study concentrated on complex referential constructions in two child–dog play sequences. It showed that the child enacted multilayered semantic configurations through indexical embodied means. The dog participated in constructing the referential link when she was asked to choose between alternatives. When it came to locating an absent referent, the child continued to employ indexical signs to support the abstract semantic structure, displaying awareness of the participants’ different semiotic worlds. The paper supports that while syntax is simplified, meaning structures may remain complex and become part of the play.

## Simple Summary

Humans are known to speak to animals. In this study, we wanted to find out how children use language when talking to cats and dogs. We specifically studied how things that are absent or only virtual possibilities are treated in interactions. We analyzed 19 videos where cats, dogs, and 6–12-year-old French-speaking children are shown in their everyday activities. There was more talking to dogs than to cats and more talking during a shared play activity than during petting or feeding. We also observed that children were likely to use short utterances. This may mean that they simplify their language structures. We then studied in more detail two video extracts showing a child and a dog playing. We saw that the child referred to absent objects or virtual possibilities in language and in bodily movements. When the dog was invited to choose between toys, she participated in construing referentiality with her head movements and vocalization. When she was asked to locate a lost ball, she did not give the expected response but showed signs of being ready to end the play. The paper supports that while syntax is simplified, meaning structures may remain complex and become part of the play.

## 1. Introduction

This paper investigates the ways in which human children use language within interspecies interaction. The focus is, first, on the overall features in middle-childhood-aged children’s cat- and dog-oriented speech, and then, more specifically, on the treatment of referential complexity by participants of different species within play interaction. The objective is to enhance our understanding of the role of the human language, especially its semantics, in activities shared with other species. The study furthermore sheds light on children’s awareness of the particularities of referential meaning construction in their companion animals and the ways in which animals participate in the multilayered semiotic processes.

### 1.1. Human–Animal Semiotics and Referentiality

The paper is grounded in the knowledge provided by recent linguistic and conversation analytic studies on language structures and use as well as sequence organization in human–animal interactions. These studies form a subfield of a larger framework of interspecies pragmatics, at the crossroads of several theoretical approaches and methodologies in linguistics. The shared object of interest is language used for talking to, for and about animals [1,2].

Human–animal interactions are organized in a way that is to a certain extent comparable to human-to-human conversations [3,4,5,6,7]. For example, they are formed of turns and sequences as constitutive units. Human and animal participants treat each other’s bodily, vocal, and linguistic behavior as interactionally meaningful, and activities they are engaged in can be mutually coordinated. It should be underlined that these features are not to be taken as manifestations of anthropomorphism, where humans would treat animal actions metaphorically as if they were produced by a human interlocutor. Turn-taking behavior and multimodality, i.e., construing meaning through several semiotic resources, have been observed in the social interaction of many species [8,9]. The sequence organization combining multiple expressive modes found in human–animal interactions is thus founded on structures of social life shared across species.

Experimental studies in ethology have addressed questions relative to those socio-cognitive capacities that make it possible to achieve social actions across species. Between so-called companion species, such as humans and cats or dogs, a common history of shared activities and close physical contacts have resulted in interspecies communication systems [10,11,12]. Within these, humans and animals display sensitivity to each other’s vocal and linguistic productions, as part of the overall embodied interaction.

Both cats and dogs are able to recognize human emotions based on visual and auditory perception [12,13,14]. Cats use vocal cues to identify and localize familiar humans [15,16]. They recognize relevant human vocabulary, e.g., their own name [17] and the name of those conspecifics with whom they share their daily lives [18]. Dogs distinguish the human language they are familiar with from other languages [19]. Human prosody is thus a relevant interactional cue for both cats and dogs. Humans, on the other hand, are able identify emotional states in other species based on vocalizations (e.g., barking and meowing) [20,21,22]. In all cases, vocal productions are best understood in heterospecific communication when combined with other expressive modes [23,24].

The modalities of interaction are likely to depend on the other participant’s species, at least partly. Children have been observed to respond with different patterns according to the kind of animal they were interacting with [25]. Concerning interaction between cats, dogs, and humans, mutual visual attention strategies are not the same. Dogs tend to use longer eye contact toward their human interlocutor, whereas cats display shorter glances. Children respond to these with different visual attention patterns [26].

Cats, dogs, and humans display adaptations in their communicative practices in interspecies interactions. For cats and dogs this entails, e.g., specific vocalizations or facial signals reserved for human-oriented communication [27,28,29]. In the case of human language addressed to pets, adult human speakers tend to use shorter units when they address an animal. In Mitchell’s study on adult American-English speakers and dogs, the mean utterance length was 2.4 words, and almost one third of the utterances were formed of only one word [30]. Syntactic constructions are simpler. They can, for example, be verbless, e.g., *Here Fifi!* [31]. There is also relatively a lot of repetition in the talk [30,31]. These features have been considered as making the talk more accessible to a non-human interlocutor.

In middle childhood, typically developing children have the cognitive-linguistic skills for perspective-taking and cooperative thinking. However, they are still in the process of acquiring cultural knowledge and social roles [32], which may generate differences in their pet-directed behavior, as compared to adults. To our knowledge, the complexity of children’s pet-oriented-talk and its implications for interspecies interaction have not yet been investigated. A previous study raised, however, that variation in interpersonal distance between children and dogs generated changes in children’s speech production, and that interpersonal distance was altered according to the ongoing activity type [33]. In human adults and dogs, the type of interspecies play project they were engaged in entailed proxemic adjustment [21,34]. What remains unknown is whether activity types play a role in the ways in which language is used (by adults or by children).

Certain types of referentiality are part of interspecies meaning construction. Both cats and dogs are able to associate lexical items of human languages with visually perceived entities [35,36]. When it comes to ostensive reference, both species are observed to use human hand or gaze pointing to locate objects [10,37]. This capacity is likely to depend on the amount of experience that the canine or feline individual has of interaction with humans [38]. As for animal vocalizations, it has been shown that they not only express emotional states but also convey context-specific indexical information. This is called *functional referentiality*, and it has been observed across species also in situations involving a third party or an object [39,40]. Furthermore, Cornips et al. [41] have analyzed cat’s bodily signals as conveying deictic reference.

These forms of referentiality are founded on indexical meaning construction. The referential link is construed within the spatio-temporal framework of the ongoing speech situation, based on a causal relationship between the sign and the referent. For example, pointing shows the direction that one must follow to identify the referent.

In human languages, unmotivated symbolic reference is particularly important. In this, the link between a sign and its referent is conventional [42]. This semiotic layer underlies displacement. It is the property of human language where events and entities that are not physically present in the speech situation are brought to interlocutors’ attention, e.g., absent entities or possible, past, and future events [43]. These types of signs are semantically complex in the sense that they stem from multilayered conceptual configurations. In these, more than one alternative is being observed in parallel. For example, in a negative expression, the entity being negated is implicitly present. Speakers refer to absent referents from within the ongoing situation, by construing another, alternative space and time, where the absent referent is located and within which it is to be identified.

**Figure 1:**
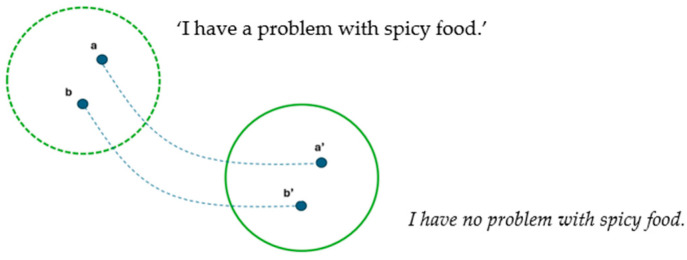
Illustration of mental spaces.

Within the framework of cognitive linguistics, Fauconnier has described this type of meaning construction with the theory of mental spaces [44,45]. Figure 1 is a simplified example of a configuration involving two mental spaces.

The mental space marked with a solid line underlies a linguistic form, for example, a negative utterance such as *I have no problem with spicy food*. It is connected to a virtual space, marked with a dashed line, which is implicitly present. In this virtual space, ‘I have a problem with spicy food’. The virtual space contains elements **a** and **b** associated with ‘I’ and ‘spicy food’. These are represented in the space underlying the linguistic form by **a’** (*I*) and **b’** (*spicy food*). Semantic structures in language lean on these conceptual connections between mental spaces. The figure is here used for illustrating what is meant by *referential complexity* and *multi-layeredness* in this paper, for example, when describing demonstrative reference to an absent entity or two alternatives. Fauconnier’s theory is not addressed further. For a more detailed account, see, e.g., [45] (pp. 44-46). It should be underlined that mental spaces are not anyone’s internal emotional or cognitive states. They are conceptual configurations, and linguistic meaning is mapped on them.

The different semiotic layers—iconic, indexical, and symbolic [42]—are in an emergent relationship to one another [46]. Humans use all three of them in human-to-human interactions, and the form-meaning relationship in many linguistic items is founded on more than one of them, in parallel [47] (For example, the imitative form *splash* is iconic, because it imitates the sound of something falling to a liquid, but it is also symbolic, since it is a conventionalized form in English for describing this sound [47]). The gradual transition zone between semiotic layers is manifest also in the fact that symbolic meaning construction has been observed in species beyond human (see, e.g., [48]). Related to this, there is experimental evidence that dogs can track human perception also in situations where it deviates from the dog’s own perception, e.g., when the human cannot see something that the dog can see [49,50]. This displays a certain degree of perspective-taking, and it also shows the capacity to treat two alternative perspectives in parallel. How these imbrications between semiotic layers are treated by participants of human–animal interactions remains unexplored.

### 1.2. Approaching Interspecies Language Use from the Perspective of Embodiment and Biosemiotics

Studying pet-oriented talk and interspecies interaction within linguistics entails a post-anthropocentric approach to language. Human language is recontextualized within a network of meaning-construction beyond human, including bodily means of expression. It is recognized as being part of the semiotic worlds of other animals, too, especially those whose everyday lives are closely entangled with human lives. These animals perceive and treat human linguistic cues in their way. This viewpoint on language is part of a larger Animal Turn in the Humanities [51,52].

Our approach is founded on Johnson’s [53] theory of embodiment in language and von Uexküll’s [54] biosemiotic model of *Umwelten*. The term *Umwelt* refers to species-typical perceptual and semiotic worlds. The two theories describe meaning construction with similar terms, although Johnson’s point of departure is human cognition and language, whereas von Uexküll is interested in ecological relations between living creatures beyond humans.

In Johnson’s words, “[t]he origin of meaning and thought is the activity of a bounded, embodied organism as it engages its various environments in ways that allow it to maintain the basic conditions for life and growth” [53] (p. 626). This interaction is conditioned by organisms’ physical properties, needs, and purposive activities. In a relationship of reciprocity, these determine the type of *affordances* [55], “patterns of meaningful perception and action”, that the organism is provided for by its environment in a specific situation [53] (pp. 626–627). In other words, affordances are what matters, what is relevant and thus meaningful for a certain species and, when considering experiences, a specific individual in a given situation. Affordances show the kind of a relationship a living being can establish with entities of the environment, for example, how to use an object [56]. In interspecies interactions, objects may be used in ways that deviate from what would be expected in human-to-human interactions [57]. The degree of complexity of the interaction and abstraction of meanings varies according to species-typical features and habitat. These fundamental embodied semiotic processes underlie even the most abstract conceptual constructions of human languages.

Jakob von Uexküll’s [54] biosemiotic theory describes meaning construction as a cyclic process between an animal and its environment. As with Gibson and Johnson, the relationship between the two is complementary here as well, in Uexküll’s terms “harmonious” [54,58]. It forms the species-typical *Umwelt* for the animal, in other words its perceptual and semiotic world. Here too, entities of the environment are more or less salient to the perceiver, depending on the connotations for action and perception that an object takes [59]. Salient entities are meaningful. Species-typical properties and life history provides the *Umwelt* with a certain structure, but it is liable to undergo changes and adaptations in interaction with the semiotic worlds of other individuals, including heterospecifics. In the history of species, *Umwelten* intertwine [60].

### 1.3. Research Questions and Hypotheses

The paper addresses the following questions:

Q1. Does the amount of children’s pet-directed speech vary according to the ongoing activity and language complexity, including referentiality?
Q2. If there is referential complexity, how is it treated by participants of different species in child–pet interaction?

Based on what we know from previous studies concerning language use, semiotic particularities, and shared activities in interspecies interactions involving human adults or children and pets, the following hypotheses are drawn up:

H1. Yes, the amount of speech varies according to the type of activity and referential complexity.
H2. In middle childhood, a human child can show their awareness of the differences between human and other-than-human semiotic worlds and tailor their expressions to make them more accessible to their animal companions.

## 2. Materials and Methods

The dataset includes 19 video recordings filmed in ecological contexts. Each video presents a child and a dog or a cat in their everyday activities, in home settings. In three of the videos the child was interacting with 2 or 3 different pets. There are a total of 11 cats and 12 dogs involved. The children were from 6- to 12-year-old speakers of French. The cats and dogs were all adults, belonged to the child’s family, and represented various breeds. Table 1 summarizes information concerning children and pets for each video.

The videos were filmed by following the child continuously for 1 h, indoors and outdoors, according to what the child chose to do. The researcher who recorded did not take part in the interactions.

The corpus was collected in 2009–2012 in Western France by M. Grandgeorge. The data gathering was conducted in accordance with the French legislation in force at that time. Being an observational study, it required no ethics committee. All pets filmed were under their owners’ responsibility during the recording. The data collection was designed in accordance with the regulations governing the use of animals for research. All parents provided free, informed written consent for the participation of their child in the study, all in accordance with the Declaration of Helsinki (6th revision), and French regulations in force at that time. The videos have been used as part of a larger dataset in previous research [26].

For the first part of the study, the aim of which was to detect variation in the children’s speech production, the following analyses were conducted: the total number of utterances per child, the number of utterances per type of activity (according to five categories: play, petting, food giving, attention getting, and others), number of words per utterance and, finally, the number of complex referential forms.

*Utterance* was defined on prosodic and functional bases as a speech unit delimited by pauses and displaying semantic cohesion. The following examples (1–5) include utterances of different length, for illustration.

(1) *ici*
  ‘here’
(2) *wouah wouah*
  ‘woof woof’
(3) *ah t’aimes bien*
  ‘oh you like that’
(4) *cherche pas y a rien à faire*
  ‘don’t even try it’s no use’
(5) *là tu sais les canards que je t’ai montrés ils étaient à moi quand j’étais petite*
  ‘and you know the ducks that I showed you they were mine when I was little’

When it comes to defining a *word*, we used morphosyntactic criteria, so that, for example, *t’aimes*, *t’ai*, or *j’étais*, in (3) and (5) each count as two words. Lexicalized interjections, such as *wouah* (2) and *ah* (3), were considered as words [30].

Each utterance was assigned an activity type tag, according to what the child–pet dyad was doing at the moment of speech: (1) play, (2) petting, (3) food giving, (4) attention getting, (5) other. (Note that we did not measure the frequency or the duration of the activity types. This prevented us from having to determine the limits of different activity sequences, e.g., whether a new play was launched or whether the ongoing activity was maintained). Activities entailing intrinsic motivation, active engagement of both participants, and relatively high physical, social, and cognitive spontaneity were considered as *play* [61]. *Petting* sequences included touching the cat or the dog with the hands, face, or feet for caressing, stroking, or kissing. *Food giving* involved serving pet food in the animal’s bowl or giving treats directly from the hands. The *attention-getting* category refers to situations where the child was seeking to establish or maintain the pet’s attention, for example, by calling the pet’s name. The category *other* covers various other types of situations, mainly those with no ongoing shared activity.

The category of *complex reference* included items that met one of the following semantic criteria:

- Demonstrative reference;
- Past- and future-time reference;
- Conditionality;
- Reference to absent entities (including wh-questions);
- Expressions of alternatives;
- Reported speech and thoughts.

A single utterance could display several types of complex reference, e.g., past-time reference and reported speech.

The second part of the study provides a more fine-grained qualitative analysis of referential complexity in interspecies language use and embodied interaction in two video extracts. Its aim is not to expose a representative specimen of all the occurrences of complex referentiality but to provide a detailed account of one of the ways in which multilayered semantic structures are integrated into child–pet interaction. The analyzed sequences were transcribed using multimodal transcription conventions [62], see Appendix A. The screen shots exposed in the paper were pseudonymized by using an image processing tool and by extracting the contours manually.

## 3. Results

### 3.1. Children’s Pet-Directed Speech

Table 2 shows the total number of utterances per child from the highest score to the lowest. The score ranges from 3 utterances/h to 292. The average is 83.7 utterances/h. Children’s age does not play a role in the amount of speech production (r = 0.251), but there is a significant difference according to sex. There are more girls above average than boys (*p* = 0.003). However, the corpus was not really designed for this question. There are fewer girls in the data than boys (8 F vs. 11 M).

The species of the animal has consequences on the amount of talk in the present data. In average, the children addressed more utterances to dogs than to cats, as shown in Table 3.

**Figure 2:**
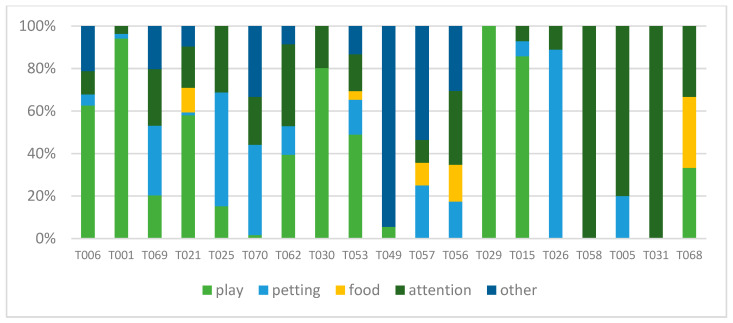
Activity types associated with talk.

We then looked at the number of utterances in view of the ongoing activity. Figure 2 presents the children from the highest score of utterances (T006, on the left) to the lowest (T068, on the right) and the proportion of five activity type categories that correspond to the ongoing action at the time of speech. The correlation between the total number of utterances and the proportion of play activity is strong (r = 0.868). Playing entailed more talking than petting, food giving or attention getting.

It should be noted, however, that play activities can take different forms. Children in videos T006 and T001 both have high scores of utterances and play activities. Yet, in the following correlation figures, we will see that they tend to display different patterns. The child–dog dyad in T001 is engaged in play where the child encourages the dog to jump over obstacles and perform other relatively goal-oriented exercises. The two bodies do not move together in these situations. When the dog is running, the child tends to be static, and the other way around. The videos T006 and T021, who both also include a rather high amount of play and talk, show children and dogs playing with a football and chasing each other. Both players run and compete spontaneously.

**Figure 3:**
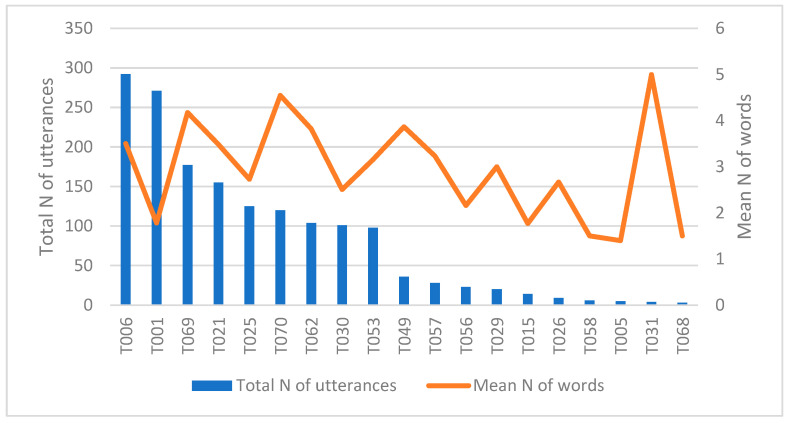
Variation in the mean number of words per utterance and the total number of utterances.

The children used in average 2.94 words per utterance, which is a slightly higher score than the one obtained by Mitchell among American-English-speaking adults with dogs (2.4 words) [30]. However, it remains remarkably lower than the mean length of utterances in the talk of non-language-impaired children in general (i.e., not specifically addressed to pets) (cf. approx. 5 words for 8-year-old English-speaking children [63]). The total number of utterances (r = 0.223) or the child’s age (r = −0.189) or sex (*p* = 0.845) did not play a role in the number of words per utterance. Figure 3 combines two measures: the variation in the mean number of words per utterance and the total number of utterances. Note the difference in the mean number of words per utterance in children’s speech in videos T006 and T001. The child in T006 displays a higher mean number of words per utterance (3.5) than the child in T001 (1.8).

**Figure 4:**
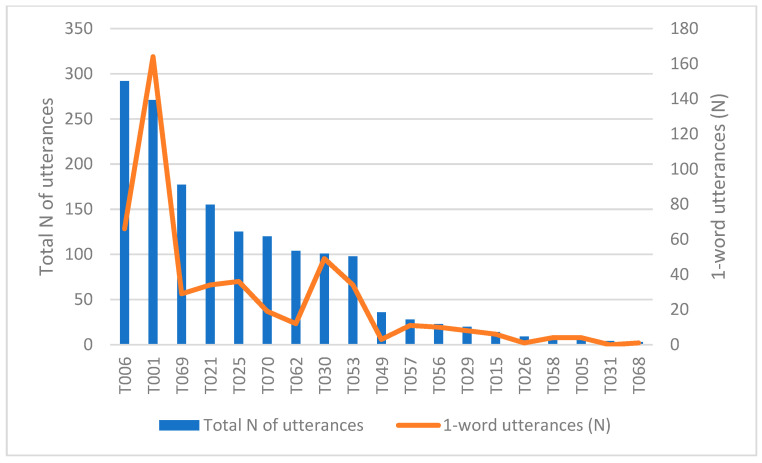
The number of one-word utterances and the total number of utterances.

About one third (33%) of the utterances are formed of only one word, which is comparable to the score raised by Mitchell (31%) [30]. The individual differences cannot be explained by the total number of utterances (r = −0.089), age (r = 0.196), or sex (*p* = 0.845) here either. Figure 4 shows the number of one-word utterances for each child, together with the total number of utterances. The difference between the children on videos T006 and T001 is again remarkable. The child in T006 displays a significantly lower percentage of one-word utterances (n = 66, 23% of all utterances) than the child in T001 (n = 164, 61% of all utterances).

**Figure 5:**
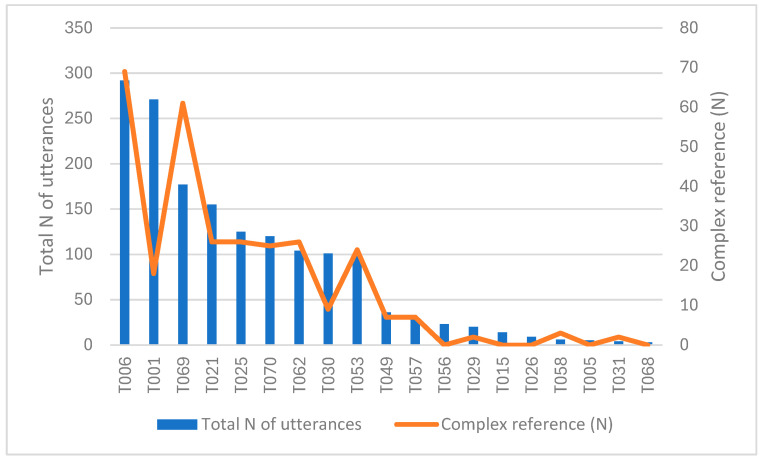
The number of complex reference items and the total number of utterances.

The final step in this part of the study was to analyze the number of referential complexity in the children’s speech. The total number of complex referential items was 305, with an average of 16 items. No statistical correlation with the total number of utterances was found (r = 0.1). However, the results for videos T001 and T006 are once again very different from each other, as can be observed in Figure 5.

The second part of the study zooms into the treatment of referential complexity in the playful interaction observed in the T006 video. It shows one of the strategies available to children when dealing with the assumed differences between the participants’ semiotic worlds.

### 3.2. Referential Complexity in Play Interaction

In this section, the aim is to understand some of the reasons behind the variation in the amount of semantic complexity in child–pet interactions. We analyze two video extracts showing that complex referential configurations can become part of the play interaction. The child enacts semantic structures that are simultaneously expressed by linguistic constructions. The pet (here, the dog) responds to this embodied meaning structure. The two play sequences are extracted from the T006 video presenting Chouka, an adult French spaniel, and Gabriel, an 11-year-old human child. As seen in the previous section, Gabriel displays the highest score of utterances among all the studied children and a relatively high number of semantically complex constructions. Most of Gabriel’s speech occurred during play interaction. On the video, Chouka and Gabriel are most of the time engaged in a dynamic play involving toys (football, tennis ball, cloth toy).

In the sequence presented in Extract 1, Gabriel is approaching Chouka and raises alternately his right hand holding a tennis ball and his left hand holding a blue cloth toy. In synchrony with his hand movements, Gabriel presents the two objects as alternatives linguistically also.


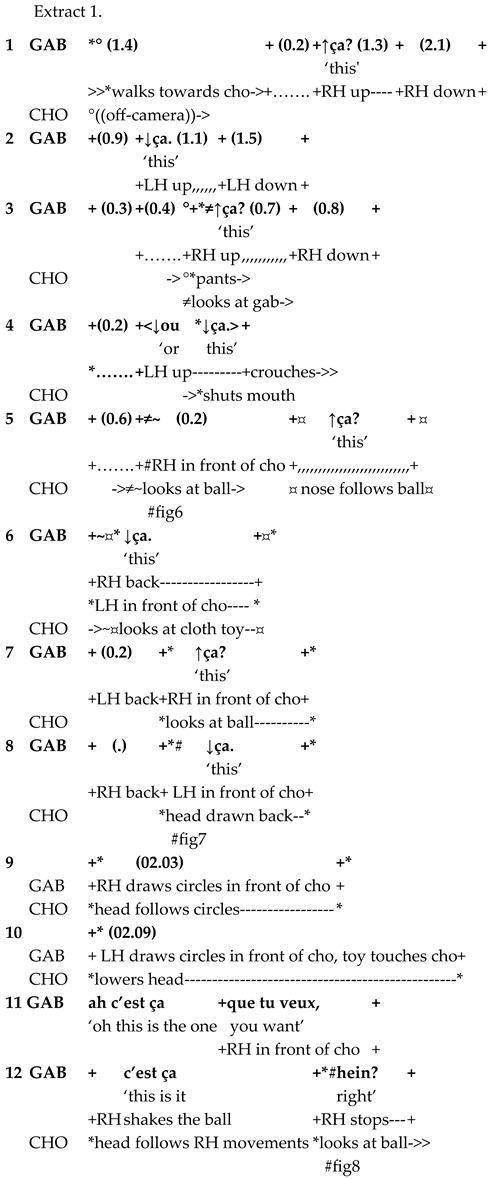
 
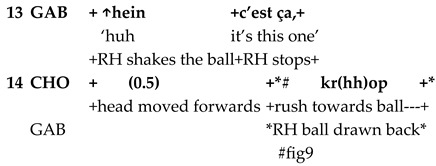



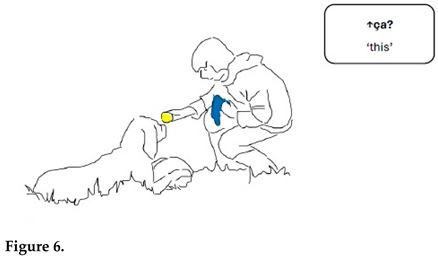



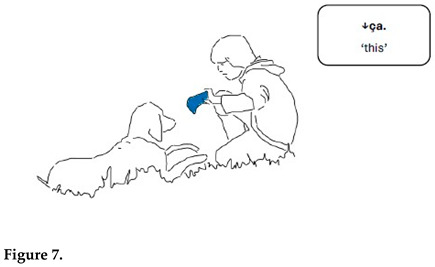



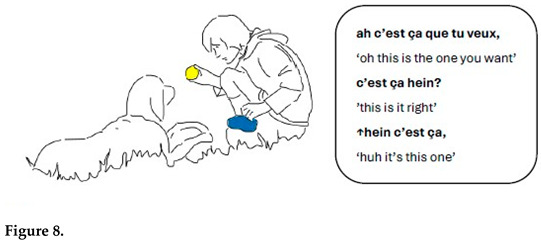



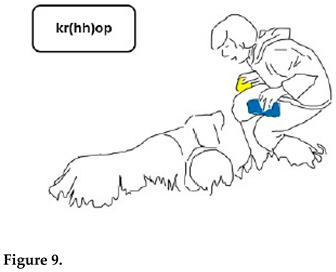


Both alternativeness and interrogative meaning are here enacted through synchronized verbal and bodily means. Reduplication in language forms and gestures construes alternativeness when Gabriel repeats the demonstrative pronoun *ça* simultaneously with hand movements. Interrogative meaning is marked with prosody (rise–fall intonation pattern). At the same time, hand movements presenting toys in alternation very close to Chouka’s face call for a reaction. The demonstrative pronoun refers alternately to the tennis ball and the cloth toy; the switch is marked with pitch and hand movements.

Gabriel treats Chouka’s gaze and head movements as a response to his questions and as an expression of the choice Chouka has made between the two objects. Chouka first alternates her gaze between the toys (lines 5–7, Figure 6). After a head withdrawal in front of the cloth toy (line 8, Figure 7), Chouka keeps her eyes on the tennis ball, even when Gabriel introduces the cloth toy again in front of her eyes (line 10). From then on, Gabriel requests confirmation of what he treats as a choice, by moving the ball in circles in front of Chouka and addressing her with syntactically more complex interrogative utterances. These are formed as cleft constructions around the demonstrative pronoun, which no longer refers to the cloth toy but only to the selected object, the tennis ball (lines 11–13, Figure 8). Chouka now tries to catch the ball by making an abrupt movement with her head towards it, simultaneously producing a sound with her mouth and, possibly, throat (line 14, Figure 9). Gabriel withdraws the ball quickly. After the sequence presented here, he validates Chouka’s choice by throwing the ball and thus pursuing the play.

After this exchange, Chouka and Gabriel continue playing with the tennis ball for approximately one minute. Gabriel then approaches Chouka again from in front of her, inquiring about the tennis ball, which is now an absent referent, see Extract 2.


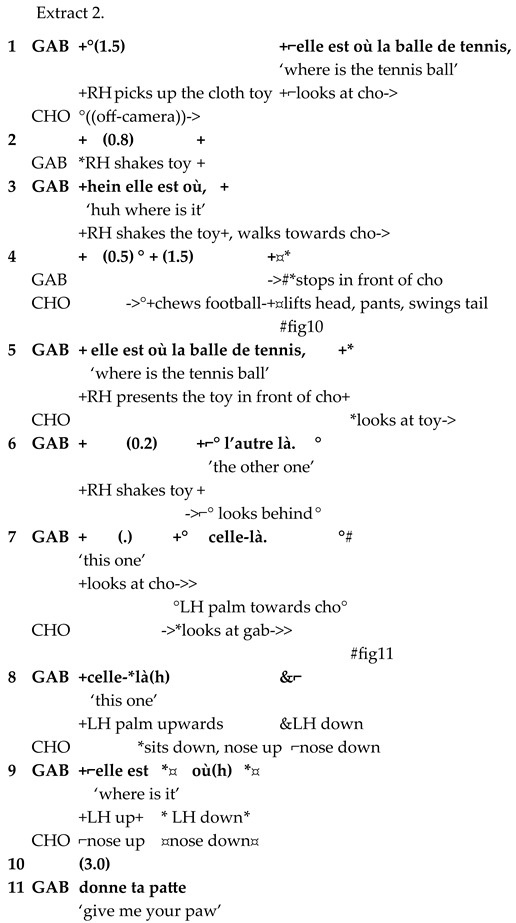



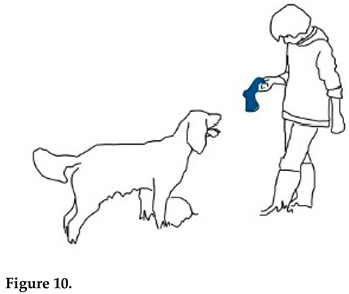



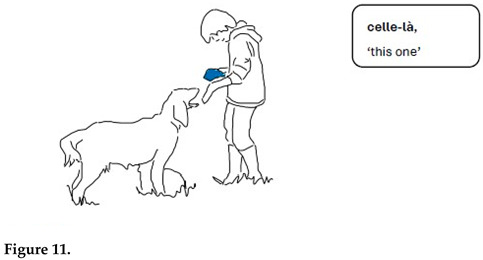


Gabriel moves from one semiotic layer to another, as he construes reference to an absent entity. The NP *balle de tennis* ‘tennis ball’, occurring in two of the three questions Gabriel addresses to Chouka (lines 1 and 5), conveys a symbolic meaning. It possibly belongs to the type of human vocabulary that Chouka identifies because of its relevance to her, together with prosodic cues (see [33]). Chouka shows her engagement in the interaction when she stops the ongoing action (chewing) and looks at the toy (line 4, Figure 10).

The referential link construed by Gabriel in what follows is founded on the semantic structure of negation. He employs the pronoun *l’autre* anaphorically to convey the meaning ‘not this’ (line 6). The semantics of *l’autre* stems from a conceptual configuration where a contrast is established between the referent and another entity [64] (pp. 133–137). In terms of mental spaces, this other entity is located in a virtual mental space that must be taken into consideration to understand the referential link construed in the linguistic form. When Gabriel shakes the toy in front of Chouka, he gives a physical form to this virtual entity that is excluded from the referential link. The demonstrative item *là* indicates that the referent cannot be located in the speaker’s sphere of perception. It is to be identified on the basis of clues that are not available in the situation of interaction [65,66]. Gabriel indicates the meaning of ‘elsewhere’ by interrupting eye contact with Chouka and looking behind him (line 6).

Gabriel’s following turns also lean on the semantics of exclusion. He shows his empty left hand and points to what is not there with the demonstrative pronoun *celle-là* ‘this one’ (lines 7–8, Figure 11). The referent is expected to be identified on the basis of what was in the hand in the preceding situation. However, the tennis ball was in the right hand then (see Extract1). In other words, the empty hand is not purely indexical, in the sense that the relationship between the missing ball and the hand that held it a minute ago is altered. The empty hand becomes a sign of absence on a level that can be seen as a first step towards symbolic meaning structure. Chouka does not give Gabriel the expected response. She follows Gabriel’s hand movements with her head, keeping her eyes on Gabriel, and finally sits down. The gaze, which no longer follows the toy or the hand, and the change in body position can be signs of her expecting Gabriel to change into another play project. This is what happens when Gabriel, after a pause (line 10), asks Chouka to give him her paw (line 11).

The analysis presented in this section showed how complex referential links are enacted by the child through linguistic and bodily means and integrated into the play activity. In the first extract, the dog’s embodied actions were treated as construing the frame within which the demonstrative reference is identified. The dog’s gaze, movements, and mouth sound formed a response to the child’s alternative questions. The ball was in this way selected as the referent of the linguistic demonstrative forms.

The second extract showed that the two players not only considered each other’s turns as meaningful components in the interaction but were also able to identify the entities their playing companion referred to through bodily and vocal pointing, when the playing project entailed choosing between alternatives. When it came to identifying an absent object, a more symbolic layer in Gabriel’s language use was activated. Gabriel showed his awareness of the different semiotic worlds of the two interlocutors by enacting the reference to the absent entity again through linguistic and bodily means. In doing so, he approached the conceptual level that had led to a successful outcome in the previous part of the play (see Extract 1). He supported the semantically complex structure with an indexical sign by showing an empty hand. The expression was tailored so as to conform to the dog’ assumed semiotic world. Chouka did not, however, provide the expected answer, showing instead signs of the play project coming to an end. Showing sensitivity to this, Gabriel switched into another project too.

## 4. Discussion

This study aimed to fill a gap in linguistic research on human–animal communication concerning interactions between children and pets. It addressed the following questions:

Q1. Does the amount of children’s pet-directed speech vary according to the ongoing activity and language complexity, including referentiality?
Q2. If there is referential complexity, how is it treated by participants of different species in child–pet interaction?

**Hypothesis** **1:** *Yes, the amount of speech varies according to the type of activity, and referential complexity.*

The hypothesis was partly confirmed. Children with a high score of utterances also displayed similarity in activity types, namely an important proportion of play interaction. However, neither the number of words per utterance, which could speak of a higher or lower degree of syntactic complexity, nor the amount of referential complexity showed any statistically significant patterns in the overall dataset. The difference observed between children with the highest number of utterances and a high proportion of play interaction suggests that a more detailed analysis of the correlation between specific play projects and referential complexity is needed, and that the extent to which the players engage in bodily synchronized movements may play a role.

**Hypothesis** **2:** *In middle childhood, a human child can show their awareness of the differences be-tween human and other-than-human semiotic worlds and tailor their expressions to make them more accessible to their animal companions.*

The hypothesis was confirmed. In the analyzed interaction, complex referential semantics, namely selecting a referent between two alternatives and pointing thereafter to an absent referent, became part of the play project. The child used rhythmically alternating hand movements that were punctuated with linguistic demonstrative items. The dog followed the child’s enactment, showing preference for one of the alternating objects. When the referent, after this, became absent, and the symbolic meaning construction was activated, the child sought to support it with the same indexical semiotic level as in the previous phase of the play, by using empty hands as signs of absence. The dog did not give the expected response, and showed inclination to end the ongoing play project, which the child finally aligned to.

The child–pet interactions investigated in this paper showed similarities with Mitchell’s [30] data including adult speakers of American English and dogs. The French-speaking children also used shorter utterances than would be expected at their age in human-to-human interaction. One third of their utterances were formed of one word only. The present dataset furthermore generated new perspectives for research concerning the ways in which not only the general activity type but also the bodily engagement of the participants in it could convey more or less complex language use.

The fine-grained analysis of two child–dog play extracts made manifest the imbrications between semiotic layers, and how the two participants treat them within the play. It also brought to the surface the embodied roots of abstract semantics, namely referential complexity, when the child enacted the indexical reference to entities that were, on a conceptual level, placed in the virtual mental space.

Human language has developed within the human *Umwelt*, but this perceptual and semiotic world has evolved in continuous interaction with the worlds of other species. Therein, human language does not belong to just humans but to the shared life world, which is not limited to human experience of the environment [67]. Just as the meaning of vocalizations and bodily expressions of other species are interpreted by humans, the different semiotic layers of human language generate meaning to animals beyond human, especially those who live in close contact with our species. The present analysis provides support for observations according to which human–animal interaction modalities depend on the species of the non-human animal. These modalities are present already in interactions involving middle-childhood-aged children. A previous study conducted on the same video corpus showed that children and dogs display longer mutual eye-contact than children and cats, who tend to use shorter glances [26]. Pet-oriented language use follows a similar pattern. Children produced more dog-oriented utterances than cat-oriented utterances. This does not mean that interactions between cats and children would be less meaningful than those involving dogs. They appear to lean on different interactional practices, however.

Prosody and lexical items are known to be relevant cues for both cats and dogs. The present study supports that even complex semantic structures are not excluded from child–pet interaction. They can be integrated within the play project through enactment. Recognizing the embodiment of human language not only opens perspectives to meaning making beyond humans and to the diversity of non-linguistic meanings in humans [53] but also reveals how such abstract conceptual configurations as displacement and mental spaces are grounded on bodily behavior.

## 5. Conclusions

In this paper, the aim was to enhance our understanding of how human children talk to pets. We first investigated the amount of pet-oriented talk, the activities during which children spoke to animals, and the length of their utterances. The analysis showed that children tend to speak more to dogs than to cats. We found a strong correlation between play activity and the amount of talk. We also observed that, just like adults in previous literature, children tend to use shorter utterances when talking to pets. This may indicate syntactic simplification, but it does not necessarily entail simple semantics. The second part of our study showed that, at least in certain types of activities, complex referential meaning structures can indeed be found. The observed child showed awareness of the differences in the semiotic worlds of the two participants, canine and human, and enacted the complex configuration through embodied means. The dog participated in constructing and confirming the referential link, in the context of selecting between alternatives. When it came to identifying and locating an absent referent, the child continued to employ indexical signs to support the potentially abstract semantic structure. More research is required to determine whether the high amount of referential complexity is connected to playful activities where the two bodies move together spontaneously, possibly competing, hiding, and searching.

## Figures and Tables

**Table 1:** Information on the filmed children and pets. (N/A = information not available).

Video	Child/ Sex	Child/Age (yrs)	Pet/ Species	Pet/Breed	Pet/Sex
T001	F	11	1 dog	Boxer	F
T005	F	6	1 cat	Domestic shorthair cat	M
T006	M	11	1 dog	French spaniel	F
T015	M	9	1 dog	Cavalier King Charles	M
T021	M	8	1 dog, 1 cat	Boxer, N/A	M, N/A
T025	F	12	1 cat	Angora	F
T026	M	8	1 cat	Domestic shorthair cat	M
T029	M	9	1 cat	N/A	M
T030	F	7	1 dog	Newfoundland	M
T031	M	7	1 dog	Mixed breed	M
T049	M	7	1 dog	Rottweiler	M
T053	F	11	2 cats	Domestic shorthair cat	M
T056	M	9	1 dog	Groendale	M
T057	M	12	1 dog	Groendale	M
T058	M	11	1 dog	Golden retriever	F
T062	F	6	1 dog	Lhassa Apso	M
T068	M	10	1 cat	Siamese	M
T069	F	7	1 dog, 2 cats	Lhassa Apso, Chartreux	F, N/A
T070	F	11	1 cat	Domestic shorthair cat	F

**Table 2:** The total number of utterances per child (N = 1591). (N/A = not applicable).

Video	Child/ Sex	Child/ Age (yrs)	Cat/ Utterances	Dog/ Utterances	Total N of Utterances
T006	M	11	N/A	292	292
T001	F	11	N/A	271	271
T069	F	7	160	17	177
T021	M	8	19	136	155
T025	F	12	125	N/A	125
T070	F	11	120	N/A	120
T062	F	6	N/A	104	104
T030	F	7	N/A	101	101
T053	F	11	98	N/A	98
T049	M	7	N/A	36	36
T057	M	12	N/A	28	28
T056	M	9	N/A	23	23
T029	M	9	20	N/A	20
T015	M	9	N/A	14	14
T026	M	8	9	N/A	9
T058	M	11	N/A	6	6
T005	F	6	5	N/A	5
T031	M	7	N/A	4	4
T068	M	10	3	N/A	3

**Table 3:** Distribution between cat- and dog-oriented utterances.

	Cats	Dogs
Total N of individuals	11	12
Total N of utterances addressed	559	1032
Mean N of utterances addressed/individual	50.8	86.0

## Data Availability

The data presented in this article are not available because of data protection constraints. The raw data supporting the conclusions of this article will be made available by the authors on request.

## Appendix A

Transcription conventions

Adapted from Harjunpää [5] and Mondada [60].

Talk	
.	falling final intonation
?	rising final intonation
,	level final intonation
**↑/↓**	rise / fall in pitch
<speak>	slow pace
(h)	breathiness
(.)	micropause
(0.5)	pause in seconds
(( ))	transcriber’s commentary
Embodied conduct	
* * / + + / ° °	Descriptions of embodied movements are delimited between
	two identical symbols and are synchronized with corresponding
	stretches of talk/lapses of time.
->	Action continues across subsequent lines until next identical symbol (->*).
>>	Action begins before the excerpt.
->>	Action continues after the excerpt.
…..	preparation
------	apex
,,,,,,,,	retraction
fig, #	screenshot and its timing
RH	right hand
LH	left hand
